# Supplementary material for: Identification of Follow-Up Markers for Rehabilitation Management in Patients with Vestibular Schwannoma
Source: J Clin Med. 2023 Sep 13;12(18):5947. doi: 10.3390/jcm12185947 (PMC10531600; doi:10.3390/jcm12185947)
Supplement: Supplementary file 1 [file jcm-12-05947-s001.zip › jcm-2505430-supplementary.pdf]

Identification of follow-up markers for rehabilitation management in patients with vestibular schwannoma

Table S1 : KANZAKI classification

Grade	Description
I	Intracanalicular
IIa	Extending 10 mm or less into the posterior fossa
IIb	Extending 11-20 mm into the posterior fossa
III	Extending 21-30 mm into the posterior fossa
IV	Extending 31-40 mm into the posterior fossa
V	Extending 41 mm or more into the posterior fossa

Table S2 : AAO-HNS classification

Class	4PTA3 (db)	Speech discrimination (WRS %)
I	0-30	70-100
II	31-50	50-69
III	>50	100-50
IV	0-100	<50

Legend. 4PTA3: (Pure Tone Hearing Loss) average of the 500, 1000, 2000 and 3000 Hz airway thresholds; WRS: (Word Recognition Score) best score obtained up to a level of SRT + 35 dB or the maximum speech comfort level. If the PTA class does not match the WRS class, it is not specified which class to use. We have opted to apply the recommendations made for the Tokyo classification.

Table S3: Video Nystagmography (VNG) data and standards

Rewiew phase	Test	Unit	Normative references
**Calibration**	*L*	ms	< 280 if L increases, aspecific central involvement
	V	°/s	> 380 if lower: brain stern origin ?
	P	%	[80 ; 105] if dysmetria: cerebellar origin?
**VNG Kinetic**	VVOR	GAIN	[0.8 ;1.1] and
		PREP °/s	< 2
	VOR, VOR 2	GAIN	[0.4 ; 1] and
		PREP °/s	< 2
	OFI	GAIN	≤ 0.1 or
		PREP °/s	Near 0
**VNG Thermal**	PA	°/s	< 2 and
	Reflectivity	°/s	[20 ; 80] and
	Deficit	%	< 30

Legend: L: latency; V: velocity; P: precision; °/s: degrees per second; %: percentage; ms: millisecond; PREP: preponderance; VVOR: visual-vestibulo-ocular reflex; VOR: vestibulo-ocular reflex; VOR2: vestibulo-ocular reflex in double task; OFI: index of ocular fixation; PA: absolute preponderance.

Table S4: Data and standards of the Multitest® posturography

Conditions	*Description of the conditions*	*Evaluation parameters*	*Normative references:*							
			Age		20-30	30-40	40-50	50-60	60-70	70+
			STABLE EO	nb-EO	54	54	61	52	56	56
				mean-EO	0.75	0.72	0.84	0.94	1.11	1.31
				sd-EO	0.44	0.44	0.49	0.61	0.59	0.58
1	STABLE EO			born_inf (2sd)-EO	-0.14	-0.16	-0.14	-0.28	-0.08	0.14
2	STABLE EC	PII		born_sup(2sd)-EO	1.64	1.60	1.82	2.16	2.30	2.48
3	STABLE OKN	Abnormal if	STABLE EC	nb-EC	54	54	61	52	56	56
4	UNSTABLE EO	above age		mean-EC	0.91	0.93	1.01	1.13	1.22	1.59
5	UNSTABLE EC	category		sd-EC	0.44	0.63	0.57	0.58	0.58	0.65
6	UNSTABLE OKN	standards		born_inf (2sd)-EC	0.02	-0.32	-0.14	-0.03	0.06	0.29
				born_sup(2sd)-EC	1.80	2.19	2.16	2.29	2.39	2.89
			STABLE OKN	nb-OKN	54	54	61	52	56	56
				mean-OKN	1.66	1.76	1.95	2.14	2.51	2.86
				sd-OKN	0.89	0.96	1.08	0.82	0.85	1.03
				born_inf (2sd)-OKN	-0.13	-0.16	-0.21	0.50	0.81	0.80
				born_sup(2sd)-OKN	3.45	3.67	4.11	3.78	4.21	4.92
All		FALL	None in the 6 conditions							
(3+6)/(2+5)		VD	Near 0							

Legend: EO: eyes open; EC: eyes closed; OKN: optokinetic; VD: visual dependence; PII: postural instability index; SD: standard deviation.

Table S5: Subjective visual vertical (SVV) data and standards

Conditions	Unit	Normative values
Static	°	[-2.5 ; 2.5]

Legend : ° : degree

Table S6 : le Dizziness Handicap Inventory (DHI)

Scale	Number of objects	After recording, number of each item								
Physical (P)	7	1	4	8	11	13	17	25		
Emotional (E)	9	2	9	10	15	18	20	21	22	23
Functional (F)	9	3	5	6	7	12	14	16	19	24

Conversion steps: The elements are recoded according to the following conditions yes = 4, sometimes = 2 no = 0; the scores are averaged per dimension. In fine, each dimension is plotted as a % with maximum scores per dimension P=28; E= 36; F=36.

Table S7: Le Penn Acoustic Neuroma Quality-of-Life (PANQOL)

Scale	Number of objects	After recording, classification by dimension and conversion of scores					
Balance	6	4	5	6	7	8	9
Hearing	4	1	2	3	20		
Anxiety	4	1	2	3	20		
Energy	6	13	17	21	22	23	24
Pain	1	14					
Face	1	10	11	12			
General Health	2	25	26				

Conversion steps: Items are recoded as follows: 1 = 0; 2 = 25; 3 = 50; 4 = 75; 5 = 100 except for item 25: 1 = 100; 2 = 75; 3 = 50; 4 = 25; 5 = 0. Once the average per dimension has been established, conversion into % using the following score conversion formula: [Actual domain score - Minimum possible domain score] / [Maximum possible domain score - Minimum possible domain score].

Table S8: Le Short Form (36) health survey (SF 36)

Scale	Number of objects	After recording to table 1, average the following elements									
Physical functioning	10	Q3a	Q3b	Q3c	Q3d	Q3e	Q3f	Q3g	Q3h	Q3i	Q3j
Role limitations due to physical health	4	Q4a	Q4b	Q4c	Q4d						
Role limitations due to emotional problems	3	Q5a	Q5b	Q5c							
Energy / fatigue	4	Q9a	Q9e	Q9g	Q9i						
Emotional well-being	5	Q9b	Q9c	Q9d	Q9f	Q9h					
Social Functioning	2	Q6	Q10								
Pain	2	Q7	Q8								
General health	5	Q1	Q11a	Q11b	Q11c	Q11d					
Declared health transmission	1	Q2									

Conversion stage: Items are recoded according to the 1992 interpretation manual. Once the average of each dimension has been established, conversion into % is carried out using the following formula = ((Patient's average raw score - Lowest possible raw score)) / (Possible raw range score).

Table S9: Link between compliance and non-compliance status between the 4 variables on which the standards are defined

Link studied	Link coefficient
**VVOR et VOR**	0.291
**VVOR et VOR2**	0.262
**VVOR et OFI**	0.052
**VOR et VOR2**	**0.734**
**VOR et OFI**	0.135
**VOR2 et OFI**	0.207

Reading key: the closer the link is to 1, the stronger it is and the more this means that the change in one of the elements is accompanied by a change in the other element.

Legend: VVOR: kinetic burst test of the visuo-vestibular-ocular reflex, VOR: kinetic burst test of the vestibulo-ocular reflex, VOR2: double-task test of the vestibulo-ocular reflex, OFI: kinetic burst test with ocular fixation.

Table S10: Breakdown of workforce according to whether they have standardised thermal VNG indicators for periods T1, T2, T3

	**Absolute preponderance**		**Reflectivity**		**Deficit**	
	**n**	**%**	**n**	**%**	**n**	**%**
**T1**	20	**62.5**	23	**72.0**	8	**25.0**
**T2**	10	**31.2**	4	**12.5**	0	**00.0**
**T3**	19	**59.4**	17	**53.1**	0	**00.0**

Reading key: at T1, 10 out of 32 patients had an absolute preponderance within the norms, 4 had reflectivity within the norms and none had a normalised deficit.
